# Psychometric Properties and Measurement Equivalence of the Workplace Incivility Scale Across Cultures: Insights from Daily Diary Study

**DOI:** 10.1177/00332941241293669

**Published:** 2024-10-16

**Authors:** Mujahid Iqbal, Maryum Bibi, Yu Yan, Shuai Yuan, Sumaira Mubarik

**Affiliations:** Department of Psychology, School of Advanced Interdisciplinary Studies, 12390 Wuhan University, Wuhan, China; School of Psychology, 47821 Northeast Normal University, Changchun, China; Department of Psychology, School of Advanced Interdisciplinary Studies, 12390 Wuhan University, Wuhan, China; PharmacoTherapy, Epidemiology and Economics, Groningen Research Institute of Pharmacy, 3647 University of Groningen, Groningen, The Netherlands

**Keywords:** Workplace incivility scale, measurement equivalence, cross-cultural, daily dairy, confirmatory factor analysis

## Abstract

Despite extensive research on workplace incivility, little attention has been given to its validation in culturally distinct contexts such as China and Pakistan. This study aims to bridge this gap by investigating the psychometric properties of the workplace incivility scale within these cultural frameworks. Additionally, it seeks to explore the cross-cultural measurement equivalence of the workplace incivility scale between these two diverse settings using a daily diary study design. The sample consisted of employees from service-providing organizations in China and Pakistan. Data was collected through snowball sampling, resulting in responses from 100 Chinese and 115 Pakistani employees over 10 consecutive workdays. Analysis of the daily responses, encompassing 748 from Chinese and 833 from Pakistani workers, indicated successful validation of the scale within both populations. This demonstrates the transferability of the concept of incivility to these cultural contexts. An integral aspect of this study is the consideration of potential cultural variations in workplace uncivil behavior. The validation and measurement equivalence of the scale for the core construct serves as essential infrastructure supporting such research endeavors.

## Introduction

Workplace incivility refers to “low-intensity deviant behavior with ambiguous intent to harm the target, violating workplace norms for mutual respect”, and represents a subtle manifestation of workplace violence (Andersson & Pearson, 1999, p. 475). Characteristics of workplace incivility include interruptions, receiving angry outbursts, and being subjected to hostile looks from coworkers and supervisors (Cortina et al., 2022). Currently, workplace incivility is believed to be more prevalent than other forms of mistreatment (Dhanani et al., 2021), and as such, it may have detrimental implications for both employees and organizations (Cortina et al., 2022; Han et al., 2022).

Workplace incivility is a global issue among the Western, and Asian cultures (Chen et al., 2019; Iqbal et al., 2021; Loh et al., 2021; Schilpzand et al., 2016). Porath and Pearson (2013) reported that 98% of workers encounter incivility in the workplace, with 50% experiencing such behavior on a weekly basis. Incidents of workplace incivility can lead to project delays and cognitive distractions for affected employees, resulting in estimated monetary costs of $14,000 per employee annually (Porath & Pearson, 2013). Experiencing workplace incivility can have direct negative consequences such as turnover intention (Eissa et al., 2020), diminished psychological well-being (Holm et al., 2022), burnout (Lu et al., 2023), and psychological distress (Labelle-Deraspe & Mathieu, 2024). Schilpzand et al. (2016) emphasized the importance of continued research efforts aimed at enhancing our understanding of workplace incivility.

Instruments for assessing workplace incivility have undergone development over time. The widely used instrument in this domain is the workplace incivility scale (WIS), which comprises seven items and assesses the frequency of encountering uncivil behavior from supervisors or coworkers over the past five years (Cortina et al., 2001; Schilpzand et al., 2016). Building upon this, Cortina et al. (2013) expanded and refined the scale to include 12 items, addressing experiences such as interruptions, being targeted with angry outbursts, or receiving hostile looks from coworkers or supervisors within the past year. However, recent literature indicates that short-term (daily level) assessments of uncivil behavior are more accurate and effective than long-term assessments (Iqbal et al., 2023; Tremmel & Sonnentag, 2018). Furthermore, affective events theory (Weiss & Cropanzano, 1996) shows negative incidences at work like workplace incivility have immediate repercussions on the emotional well-being of employees.

Recently, researchers have expressed an interest in studying the short-term effects of negative work experiences (Blanco-Donoso et al., 2019; Chong et al., 2020; Tremmel & Sonnentag, 2018). Since incivility is recorded daily and weekly (Chong et al., 2020; Porath & Pearson, 2013; Shin et al., 2021), it is logical to assume that targets’ experience differs from day to day. The researchers are still studying how this daily change in perceived workplace incivility affects targets, especially their short-term reactions. Because incivility is considered a moderate type of workplace mistreatment, it is not clear if it might cause short-term affective responses. As a result, we think that a daily diary design can help us better understand the short-term effects of daily workplace incivility on our study participants.

Furthermore, the existing literature predominantly focuses on research into workplace incivility within Europe and North America, which represent only a 27% portion of the global population (Triandis & Gelfand, 1998), with limited attention given to studies conducted in Asia (China and Pakistan), particularly regarding the adaptation and development of scales to measure workplace incivility. It is essential to differentiate between workplace incivility and tolerance to workplace incivility, which refers to the extent to which individuals endure or accept uncivil behaviors without necessarily reporting them. Understanding this distinction is vital for both researchers and practitioners, as it can shape interventions and policies aimed at mitigating the negative effects of workplace incivility (Abubakar, Megeirhi, et al., 2018; Abubakar, Yazdian, et al., 2018; Megeirhi et al., 2020). Tolerance can influence employees’ reactions and their overall workplace behavior, thereby contributing to a nuanced understanding of workplace dynamics.

Cross-cultural research on workplace incivility between Chinese and Pakistani workers is essential due to the geographical proximity and increased interaction resulting from initiatives like the China-Pakistan Economic Corridor (CPEC). With shared borders and growing economic ties, workers from both countries are increasingly compelled to collaborate and interact (Ali, 2020). Consequently, understanding the cultural nuances and differences in workplace behavior becomes crucial to fostering harmonious and productive work environments. China and Pakistan represent two vastly different cultural contexts, characterized by distinct societal norms, values, and communication styles (Hofstede, 2018; Hofstede & Bond, 1988). Exploring workplace incivility in these diverse settings allows for a comprehensive understanding of how cultural factors influence behavior and perception. In an era of globalization, multinational companies often employ workers from various cultural backgrounds (Gelfand et al., 2006).

This study aims to bridge this gap by investigating the applicability of the workplace incivility scale (WIS) within these cultural frameworks. The lack of validated measures in these cultures underscores the necessity for cross-cultural examination. Through the utilization of a multilevel confirmatory factor analysis (MCFA) and a diary study design, this research addresses two main objectives: (1) examining the psychometric properties of WIS within the cultural landscapes of China and Pakistan, and (2) exploring the cross-cultural measurement equivalence of the WIS between these two cultural settings.

## Material and Methods

### Procedure and Sample

The sample for this study was recruited from two Asian countries, China and Pakistan, after receiving approval from the first author’s university ethics board committee. Participants were recruited from a variety of service-oriented sectors, including academia, banking, and healthcare, which are frequently exposed to workplace incivility due to the high level of interpersonal interactions inherent in these fields (Cortina et al., 2022). To ensure a representative sample, participants had to be full-time employees whose roles required regular interaction with coworkers. A snowball sampling method was used in both China and Pakistan to facilitate recruitment across diverse organizational settings. In China, employees were recruited from public and private organizations, while in Pakistan, participants were primarily recruited via social media platforms and professional networks. This approach allowed for the inclusion of a broad range of experiences across different organizational cultures.

Data collection was conducted over 10 consecutive working days using a daily diary method. Each day, participants completed a short survey assessing their experiences with workplace incivility. This repeated-measures design allowed for a more accurate assessment of workplace behaviors, as participants were less likely to alter their responses over time due to social desirability bias (Tremmel & Sonnentag, 2018). Additionally, we assured participants that their responses were both anonymous and confidential. All participants were assigned unique identification numbers to prevent any direct link between their responses and personal identifiers. Given the sensitivity of workplace incivility as a research topic, additional precautions were taken to promote honest responses. We designed the survey to allow participants to answer comfortably and without fear of identification. They were informed that they could withdraw from the study at any time. These measures were explained clearly to ensure that participants felt safe and secure in sharing their experiences. Data was collected from May to November 2021. Our participants had various organizational positions (e.g., bank managers, cashiers, recovery officers, account managers, doctors, nurses, psychologists, lecturers, and professors).

### Chinese Sample

A total of 100 full-time employees in China were surveyed online. Participants were compensated ¥10 (about $1.5 each) for their participation. The first author made personal visits to a variety of public and private organizations in Wuhan, contacting site managers and informing them of our research. Willing employees were added to the WeChat group once their managers confirmed their organization’s willingness to participate. Initially, 140 volunteers were invited to join WeChat groups. The snowballing sample approach was used to enroll an additional 35 individuals. Out of 175 employees, thirty-six (36) members of the group have left without any reason. One hundred and thirty-nine persons (79.4%) responded to the general survey. The daily section of the survey was not completed by 39 employees. One hundred (71.9%) participants were included for hypothesis testing. We received 876 surveys. Individuals who completed surveys for at least two days were included, giving us 748 surveys (matching data sets) from 100 employed people.

The mean age of the participants was 31.70 years old (*SD* = 7.56), and (58.0 %) of them were female, (37.0 %) of employees represented more than 10 years of job experience and they worked on average 40.01 hours a week (*SD* = 10.03) and engaged with colleagues on average 30.40 hours per week (*SD* = 9.20), and the majority of (46.0%) were graduated from university, participants represented a variety of job titles, university and college employees (23.0%), bank employees (40.0%), and hospital employees (37.0%) as shown in Table 1.

**Table 1.** Demographic Characteristics of Chinese and Pakistani Sample.

Variables	Chinese sample (*n* = 100)	Pakistani sample (*n* = 115)
	Mean ± *SD*/*n(*%*)*	Mean ± *SD*/*n(*%*)*
**Age**	31.70 ± 7.56	32.60 ± 6.89
Daily interaction (hrs. per week)	30.40 ± 9.20	26.10 ± 12.10
Gender		
Male	42 (42.0)	52 (45.2)
Female	58 (58.0)	63 (54.7)
Education		
Under-graduate	20 (20.0)	16 (13.9)
Graduate	46 (46.0)	39 (33.9)
Post-graduate	34 (34.0)	60 (52.1)
Designation		
University employees	23 (23.0)	31 (26.9)
Bank employees	40 (40.0)	43 (37.3)
Hospital employees	37 (37.0)	41 (35.6)
Tenure		
4 month-2 years	17 (17.0)	24 (20.8)
2–5 years	21 (21.0)	27 (23.4)
5–10 years	25 (25.0)	39 (33.9)
>10 years	37 (37.0)	25 (21.7)
Work hours (per week)		
30 hours	21 (21.0)	28 (24.3)
40 hours	49 (49.0)	58 (50.4)
60 hours	30 (30.0)	29 (25.2)

### Pakistani sample

We recruited 115 full-time employees through snowball sampling. Participants were recruited via career-oriented social networking sites. They received a two-week free internet package (worth around $2 each) as compensation for their time. Initially, 97 employees submitted a baseline survey (phase 1). We requested participants to forward the survey link to friends who may also be willing to participate. Forty-four (44) additional participants were recruited in this manner. One hundred forty-one (141) people submitted a general survey along with demographics. Ten participants wrote their wrong identification numbers so we could not track their responses. Of the remaining 131 (92.9%) employees, we only kept data for individuals who completed all three questionnaires for at least two days, as necessary for multilevel analysis (resulting in the exclusion of another 16 individuals). After dropping missing, 115 (87.8%) employees were finally included in the study. We received 898 surveys. Only individuals who completed all three questionaries’ for a minimum of two days were included. After all, we reached 833 complete surveys (matching data sets) from 115 participants.

The final 115 participants were 32.60 years old on average (*SD* = 6.89), and the majority (54.7%) of them were female, (44.0%) of employees represented 10 years of job experience, and they worked on average 40.01 hours in a week (*SD* = 7.12) and interacted with coworkers on average 26.10 hours per week (*SD* = 12.10), with a post-graduate level education (52.1%), participants represented a variety of job titles, university and college employees (26.9%), bank employees (37.3%), and hospital employees (35.6%) as shown in Table 1.

### Measures/Instruments

The Chinese version of the scale was administered to the participants in China. For translation in Chinese, we employed the translation and back-translation procedure (Brislin, 1970). After the initial translation and back-translation process, the translated questionnaire was pre-tested with a small sample of individuals from the target population to assess its comprehensibility, clarity, and cultural appropriateness. Based on feedback from the pre-test, further revisions were made to the translated questionnaire to improve its accuracy and relevance to the target population (Schroder et al., 2011).

The English version instrument was administered to the participants in Pakistan. In Pakistan official language is English and employees can speak and understand the English language in such organizations (Iqbal et al., 2024; Sarwar & Muhammad, 2020). So, all participants were fluent in the English language and there was no need to translate the questionnaire into another language.

### Perceived Workplace Incivility

The eight items workplace incivility subscale adopted by (Jiménez et al., 2018) was applied to measure daily perceived incivility. In the daily survey, participants were questioned if each of the eight incivility incidents had occurred to them. One point was added to the incivility score for each checked experience. Participants were instructed to focus their answers on their last experiences today. With the stem adjusted to refer to the day-level perception of incivility (Volmer et al., 2012). For example, “Today, my coworker has ignored me or did not respect my opinion” from (1 = *strongly disagree*; 5 = *strongly agree*). In the Chinese sample, Cronbach’s Alpha (α) coefficients were .84. Furthermore, the Pakistani sample indicated an alpha reliability coefficient of .89.

### Data Analysis Approaches

Using SPSS (version 26), missing values were checked in the data, and cases with incomplete data were deleted. The demographic variables were calculated using ordinary descriptive statistics (Table 1). Cronbach’s alpha was calculated to assess internal consistency. For each item, we calculated its averages, *SD*, skewness, kurtosis, “alpha if item deleted,” and interclass correlations. Structure validity was examined using a three-factor model, and measurement equivalence of the three-factor model of the scale collectively was then tested. Next, we used Mplus (version 8) to conduct Multigroup Confirmatory Factor Analysis (CFA) using the “maximum likelihood with robust standard errors” estimator. Further, Chi-square metrics, comparative fit index (CFI), Tucker-Lewis index (TLI), Root Mean Square Error of Approximation (RMSEA), and Standardized Root Mean Square Residual (SRMR) were used to test model fit.

Then, we assessed configural, metric, and scalar measurement invariance between the Chinese and Pakistani sample. The measurement invariance examined whether the CFA models were appropriate for the two groups. Acceptable model fit was defined as CFI and TLI values above .95 (Kline, 1998), and RMSEA and SRMR values less than .08 (Steiger, 1998). Measurement invariance across groups was indicated by a ΔCFI less than .010 and ΔRMSEA less than .015 according to earlier literature (Chen, 2007).

Measurement invariance testing was primarily conducted in four steps. First, single-group CFAs were investigated for each country. Second, configural invariance with multi-group CFAs without equality constraints was examined. Third, the model was tested for weak (metric) measurement invariance with multi-group CFAs restricting all factor loadings to be equal across both samples. Fourth, the model was tested for strong (scalar) measurement invariance by restricting all thresholds to be equal across both samples. In every step, each model was accepted only when the values of CFI, RMSEA and SRMR were within the cutoff criteria. When full invariance was not found, partial weak and partial strong measurement invariance was tested by setting free specific factor loadings or thresholds (Byrne et al., 1989). Following Byrne et al. (1989), we continued partial measurement invariance until at least two items were invariant.

## Results

### Descriptive Statistics

The descriptive statistics and percentages of the total sample of Chinese and Pakistanis are given in Table 1.

### Reliability Test

Reliability results are given in Table 2. Notably, the Chinese sample exhibited lower mean scores across all items, while the Pakistani sample demonstrated higher mean scores. Internal consistency was robust in both samples, with high Cronbach’s alpha values. Specifically, in the Chinese sample, the workplace incivility scale showed a Cronbach’s alpha of .84, surpassing the desirable threshold of .80 and indicating strong internal consistency. Similarly, the Pakistani sample displayed a Cronbach’s alpha of .89 for the workplace incivility scale, further affirming its high internal consistency. These findings underscore the reliability of the scale across cultural contexts, despite variations in mean scores.

**Table 2.** Means, Standard Deviations, Skewness, Kurtosis, Corrected Item-Total Score Correlation (r_tt_), Loading, and Alpha of all Items of WIS in Chinese and Pakistani Sample.

Items	*M*	*SD*	Skewness	Kurtosis	*r*_*tt*_	Loading	Alpha if item deleted
Chinese — WIS							
1. 今天我的同事忽略我, 或者不尊重我的意见	2.32	0.81	1.30	0.94	.42	.60	.73
2. 今天我的同事八卦我或其他人	4.65	0.85	0.90	−0.86	.64	.63	.75
3. 今天我的同事讨论时不考虑我	3.06	0.87	0.79	−2.95	.69	.61	.84
4. 今天我的同事质疑我的专业能力	2.66	0.70	−1.45	0 .69	.49	.64	.78
5. 今天我的同事试图打听我的私生活	3.01	0.91	2.18	2.76	.52	.68	.76
6. 今天我的同事有令人讨厌的表情或手势	2.90	0.77	1.30	−0.56	.37	.70	.80
7. 今天我的同事消极应对/抱怨工作要求	2.70	0.79	−0.75	1.76	.59	.65	.86
8. 今天我的同事表现得很没有礼貌	3.51	0.89	0.92	1.11	.67	.71	.77
Cronbach’s alpha	.84						
Pakistanis — WIS							
1. Today my coworker ignored me or did not respect my opinion.	4.52	0.85	−0.95	1.45	.70	.74	.78
2. Today my coworker gossiped about me or my colleagues.	3.79	0.91	0.59	−0.60	.63	.67	.83
3. Today my coworker excluded me from a discussion.	2.56	0.90	1.45	−0.49	.56	.66	.87
4. Today my coworker questioned my professional competence.	4.97	0.86	−1.64	1.46	.66	.61	.76
5. Today my coworker showed inappropriate interest in my personal life.	4.03	0.92	0.68	−1.76	.54	.68	.70
6. Today my coworker showed dislike/scorn in sarcasm, facial expressions, or gestures.	5.00	0.97	0.93	0.97	.47	.73	.89
7. Today my coworker expressed negative attitudes or complained about work demands.	2.89	0.80	−2.13	0.90	.45	.75	.77
8. Today my coworker acted in a rude, inconsiderate manner.	4.75	0.84	1.34	2.72	.64	.60	.80
Cronbach’s alpha	.89						

*Note.* Level 1 *n* = 748 for the Chinese sample and Level 1 *n* = 833 for the Pakistani sample. WIS = Workplace incivility scale; *M* = Mean; *SD* = Standard deviation; Loading = Factors loading.

### Calculating the Difference in Interclass Correlations

The decision to calculate the difference in interclass correlation (ICCs) in this research is driven by the fundamental need to evaluate the stability and consistency of measurements across two distinct samples. Calculating the difference in ICCs allows for a rigorous examination of the extent to which the measurements maintain consistency across these culturally diverse settings (Perisic & Rosner, 1999).

As shown in Table 3, the differences in ICCs values for the workplace incivility scale between China and Pakistan reveal intriguing insights. For Item 1, a substantial deviation of −0.28 suggests a notable variation in the intraclass correlation between the two cultural contexts. Meanwhile, Item 2 and Item 3 exhibit moderate variations of 0.13 each, indicating some degree of difference in ICCs. Item 4 stands out with a discernible decrease of −0.17, reflecting a notable discrepancy in intraclass correlation. In contrast, Item 5 shows a substantial variation of 0.18, suggesting a noteworthy difference in measurement stability. Item 6 displays a modest variation of −0.10, underscoring the nuanced nature of ICC differences. Similarly, Item 7 demonstrates a moderate difference of 0.14, pointing to a discernible variation in ICCs. Lastly, Item 8 exhibits a substantial variation of 0.23, highlighting a notable discrepancy in measurement stability. These findings emphasize the need for a comprehensive understanding of cultural influences and their impact on measurement stability in cross-cultural research.

**Table 3.** Interclass Correlations of WIS and Difference Between Chinese and Pakistani Sample.

WIS	Interclass correlations		
	China	Pakistan	Difference
Item 1	.42	.70	−.28
Item 2	.76	.63	.13
Item 3	.69	.56	.13
Item 4	.49	.66	−.17
Item 5	.72	.54	.18
Item 6	.37	.47	−.10
Item 7	.59	.45	.14
Item 8	.87	.64	.23

*Note.* Level 1 *n* = 748 for the Chinese sample and Level 1 *n* = 833 for the Pakistani sample. ICCs values less than 0.5 are indicative of poor reliability, values between 0.5 and 0.75 indicate moderate reliability, values between 0.75 and 0.90 indicate good reliability, and values greater than 0.90 indicate excellent reliability. WIS = Workplace Incivility Scale.

### Validity Test and Cross-Cultural Measurement Invariance Analysis

The structure validity was assessed using a three-factor model, followed by testing the measurement equivalence of the scale’s three-factor model collectively. The validity of the scale and cross-cultural measurement invariance of the workplace incivility scale are presented in Table 4. The findings revealed that the model goodness of fit indices had reached acceptable values, indicating a good model fit. Results showed that the model fit for the three-factor correlated model was good. The values of the alternative fit indices showed that they were above the minimum cutoff threshold of .90, indicating that the measurement model achieved a reasonable fit of the data: For Chinese (*χ2* = 196.436, *df* = 28, *p* < .01), CFI = .96, TLI = .97, RMSEA = .05, 90% CI [.04, .07] and SRMR = .04) and for the Pakistanis (*χ2* = 179.319, df = 28, *p* < .01), CFI = .99, TLI = .98, RMSEA = .03, 90% CI [.02, .04] and SRMR = .05). The values of ΔCFI and ΔRMSEA were less than .01 which indicates measurement invariance across the two groups.

**Table 4.** Cross-Cultural Measurement Invariance Tests Result of the WIS.

Models	*χ* ^2^	*df*	*CFI*	*TLI*	*RMSEA*	*SRMR*	*∆CFI*	*∆RMSEA*
					*(90%CI)*			
Three-factor model								
Single group CFA								
Chinese	196.436**	28	.961	.971	.051 (.049, .075)	.045	—	—
Pakistanis	179.364**	28	.990	.987	.037 (.024, .044)	.056	—	—
Multigroup CFA								
Configural	147.319**	28	.980	.974	.065 (.059, .070)	.024	—	—
Metric (weak)	135.725**	28	.972	.991	.062 (.055, .077)	.015	.008	.003
Scalar (strong)	218.572**	28	.970	.987	.061 (.045, .091)	.047	.002	.001

*Note.* Level 1 *n* = 748 for the Chinese sample and Level 1 *n* = 833 for the Pakistani sample. WIS = Workplace incivility scale; χ^2^ = Fit indices of chi-square; df = Degrees of freedom; CI = Confidence interval; CFI = Comparative fit index; TLI = Tucker–Lewis index; RMSEA = Root mean square error of approximation; SRMR = Standardized root mean square residual; ΔCFI = Change in CFI relative to the preceding model; ΔRMSEA = Change in RMSEA relative to the preceding model. **p < .01.

## Discussion

The present study is the first to introduce psychometric properties of the workplace incivility scale (WIS) (adapted by Jimenez et al., 2018), and cross-cultural measurement invariance within the contexts of China and Pakistan. This study aimed to validate the WIS and test cross-cultural measurement invariance between Chinese and Pakistani employees. The primary aim was achieved by establishing and validating the reliability of WIS through a rigorous application of multilevel confirmatory factor analysis (MCFA). Additionally, the cross-cultural measurement invariance of WIS was achieved, utilizing a diary study design to understand their consistency in diverse settings between China and Pakistan (Iqbal et al., 2023). Through the accomplishment of these objectives, this study aspired to deepen our comprehension of workplace incivility in varying cultural landscapes, providing robust assessment tools applicable to efficacy in both China and Pakistan.

Exploratory factor analysis unveiled a four-factor solution for the WIS. Notably, the fourth factor encompassed three items pertaining to inappropriate inquiries into personal matters, indicative of privacy violations. An interpretation of this unexpected finding may be rooted in cultural nuances: Our study cohort comprised Pakistani employees, where there exists a prevalent cultural norm of segregating personal and professional domains. Consequently, discussions concerning personal life in the workplace might be infrequent, leading to a scarcity of inappropriate conversations about personal matters (Iqbal et al., 2021). This observation is further supported by our data, as evidenced by the relatively lower means associated with all three items concerning inappropriate interest in personal life compared to other items. However, it is noteworthy that a similar factor was identified in a study involving Japanese workers utilizing a different adaptation of the WIS (Tsuno et al., 2017), suggesting a cross-cultural recurrence rather than a culture-specific finding in our investigation. In the Japanese study, the authors proposed that discussions about personal matters could be perceived as a form of sexual harassment. It is essential to delineate between incivility and sexual harassment, as they are distinct yet correlated constructs (Lim & Cortina, 2005). While individuals experiencing sexual harassment often encounter greater incivility, experiencing workplace incivility does not necessarily imply experiencing sexual harassment (Rai & Agarwal, 2017). Therefore, it is imperative to treat both constructs independently.

The confirmatory factor analysis yielded better-fit indices for the original three-factor solution compared to the four-factor solution. However, the three-factor solution was nearly identical to an alternative model where the items associated with the unexpected fourth factor were excluded. As a precautionary measure, we recommend refraining from utilizing items related to inappropriate interest in personal life until further research is conducted. The bifactor model analysis revealed that the WIS can effectively capture feedback regarding general incivility as well as specific incivility domains (supervisor, coworker). Consequently, both the Chinese and Pakistani versions of the WIS can offer targeted feedback on various forms of detrimental behavior within organizations, facilitating the development of tailored interventions.

Notably, the Chinese sample exhibited lower mean scores across all items, while the Pakistani sample demonstrated higher mean scores. Internal consistency was robust in both samples, with high Cronbach’s alpha values. Specifically, in the Chinese sample, the workplace incivility scale showed a Cronbach’s alpha of .84, surpassing the desirable threshold of .80 and indicating strong internal consistency. Similarly, the Pakistani sample displayed a Cronbach’s alpha of .89 for the workplace incivility scale, further affirming its high internal consistency (Iqbal et al., 2023). Corrected item-total correlations demonstrated favorable results, surpassing the minimum threshold of 0.40 for every item (Zijlmans et al., 2019). Notably, lower mean values on the scale indicate that Chinese respondents tended to rate items towards the lower end of the scale, whereas higher mean values among Pakistani respondents suggest a greater prevalence of incivility experiences within the sample. This trend aligns with previous studies by (Leiter et al., 2011; Spence Laschinger et al., 2009), who also reported relatively lower mean scores for incivility in their respective samples. These findings align with previous research by Hofstede (2011), which indicated that cultural context significantly shapes workplace behavior. Specifically, Hofstede’s dimensions of culture demonstrate how variations in values, such as individualism versus collectivism, can influence interactions and expectations in organizational settings. Similarly, Gelfand et al. (2006) emphasize that cultural contexts play a crucial role in shaping employee interactions and behaviors, suggesting that organizations need to be aware of these differences to create a harmonious work environment (Gelfand et al., 2006; Hofstede et al., 2005). Future studies could delve into the role of organizational policies in mitigating workplace incivility across cultures. Leiter and Laschinger (2006) argue that effective leadership and supportive organizational policies can significantly reduce workplace incivility, fostering a more respectful and productive work environment. Moreover, Gupta et al. (2002) highlight that understanding cultural clusters and their implications can inform the development of targeted interventions and policies that cater to diverse teams (Gupta et al., 2002; Leiter & Laschinger, 2006).

The robust validation of WIS in the distinct cultural contexts of China and Pakistan is considered a significant achievement in this research. The demonstrated reliability and validity of the scale through multilevel confirmatory factor analysis (MCFA) underscore the applicability and consistency of the measurement tools across these diverse settings. The success in establishing the psychometric properties provides a solid foundation for utilizing these scales effectively in both Chinese and Pakistani organizational contexts. This outcome not only enhances the reliability of future research conducted within these cultures but also offers researchers and practitioners in organizational psychology a valuable resource for cross-cultural comparisons in the realm of workplace incivility.

Moreover, the exploration of cross-cultural measurement invariance added another layer of depth to the study’s contributions. The confirmation that the scales maintain invariance between China and Pakistan in a diary study design strengthens the generalizability of the findings. This suggests that the conceptualization and measurement of workplace incivility are consistent across these distinct cultural landscapes. The study’s success in achieving cross-cultural measurement invariance establishes a crucial foundation for comparative analyses, allowing researchers to draw meaningful conclusions about the prevalence and impact of workplace incivility in different cultural contexts (Gupta et al., 2017).

Overall, the psychometric properties of all scales demonstrated robustness in both samples. Single-group Confirmatory Factor Analyses (CFAs) consistently revealed identical factor structures for scale. This is in line with previous findings reporting good internal consistency in Canadian samples (Spence Laschinger et al., 2009; Leiter et al., 2011). Furthermore, multigroup CFAs for the scale substantiated the hypothesis of a consistent structure with equivalent loadings across groups. Latent means were systematically compared for each sample, revealing elevated levels of perceived incivility among Pakistani participants compared to their Chinese counterparts (Smidt et al., 2016).

Various factors could account for the observed variations in factor loadings between our Chinese and Pakistani samples. Firstly, there may be distinct interpretations of the constructs in each cultural context. Secondly, specific items might carry more weight in contributing to the overall score within one culture, leading to higher factor loadings for these items in the Chinese sample compared to the Pakistani sample (Kamboj & Garg, 2022). Thirdly, differences in thresholds might arise due to social norms or a social desirability effect; participants could be hesitant to admit their reactions to different situations or face challenges in relaxation. Some participants might exhibit stronger responses to certain items despite having the same factor mean, while others could have different reference points when responding about themselves (Bibi et al., 2020).

These disparities in results may be attributed to the distinct cultural landscapes of China and Pakistan, which exhibit variations in several aspects. Religion plays a significant role in shaping the workplace dynamics of Pakistani workers, with Islamic principles often influencing social interactions and decision-making. In contrast, Chinese workers are less influenced by religion in the workplace, as Confucian values tend to emphasize moral and ethical behavior rather than religious doctrines (Zeng & Hao, 2016). Additionally, language acts as a cultural bridge for communication among Chinese workers, while in Pakistan, language differences, particularly between Urdu and regional dialects, can sometimes create barriers in workplace interactions (Asif et al., 2021). Social norms in China often prioritize harmony and respect for authority, which may lead to indirect communication styles and a reluctance to address conflicts openly, potentially fostering workplace incivility when grievances remain unaddressed. Conversely, in Pakistan, hierarchical structures and collectivist values may contribute to a culture where assertive communication is less common, leading to misunderstandings and tensions that can escalate into incivility (Naeem et al., 2018). These cultural distinctions highlight the diverse influences that religion, language and social norms exert on the work environments of Chinese and Pakistani workers (Iqbal et al., 2023).

Beyond these general cultural distinctions, specific cultural nuances impact workplace dynamics. Hierarchy and respect play a significant role in Chinese workplaces, potentially affecting the reporting of workplace incivility, especially if superiors are involved. Communication styles also vary, with indirect communication favored in China and more direct approaches in Pakistan when addressing workplace issues (Naeem et al., 2020). The balance between collectivism and individualism differs, influencing responses to incivility. Chinese employees may prioritize harmony, and avoiding conflict to preserve group well-being, while Pakistanis may assert their rights more directly. The concept of “face” is crucial in China, influencing the reporting of incivility to avoid loss of face (Prosekov, 2019). In Pakistan, there might be a greater willingness to report incivility to preserve self-respect. Workplace dynamics differ in terms of conformity, conflict resolution, gender roles, and time orientation, reflecting the rich cultural diversity between China and Pakistan (Kimel et al., 2022).

### Implications

This study significantly enhances our understanding by affirming the reliability and validity of the WIS within the Chinese and Pakistani populations. The findings underscore the efficacy of the validated and culturally adapted WIS in measuring perceived workplace incivility across both countries. Notably, to our knowledge, no prior research has investigated the applicability of the WIS within organizational contexts in China and Pakistan. Therefore, measurement equivalence in our study not only fills a critical gap in the literature but also provides a solid foundation for future cross-cultural studies on workplace incivility.

The results of this study offer key lessons for organizations operating in multicultural environments. Understanding the distinct cultural interpretations of workplace incivility can inform tailored interventions. Organizations should consider implementing training programs focused on conflict resolution that are sensitive to cultural differences. For instance, workshops promoting respectful communication could enhance interpersonal relations. The validated WIS can play a crucial role in guiding organizations toward creating inclusive policies. By leveraging these findings, organizations can establish ongoing training and support systems that address the unique cultural dynamics within their workforce. Ultimately, this research contributes to the growing field of organizational psychology by highlighting the significance of cultural context in understanding workplace behavior.

By studying workplace incivility in China and Pakistan, insights gained can be applied to similar multicultural work environments globally, contributing to the development of effective cross-cultural management strategies (Earley & Mosakowski, 2004; Kirkman et al., 2006). Given historical tensions and potential misunderstandings between neighboring countries like China and Pakistan, understanding cultural differences in workplace behavior can contribute to conflict resolution and improved intercultural relations at both individual and organizational levels (Ting-Toomey & Chung, 2005; Triandis, 1994). Research findings on workplace incivility in China and Pakistan can inform the development of culturally sensitive policies and training programs aimed at promoting respectful and inclusive workplaces (Jack & Gill, 2013; Lynch & Hanson, 2011). This can ultimately enhance employee satisfaction, retention, and organizational performance (Andrade & Neves, 2022). Conducting cross-cultural research adds to the body of knowledge in the field of organizational behavior and cross-cultural psychology. It enriches theoretical frameworks and methodologies by testing their applicability across diverse cultural contexts, thereby advancing academic discourse and understanding (Hofstede, 2011; Gelfand et al., 2013).

The findings of this study underscore the necessity for organizations in both China and Pakistan to prioritize the cultivation of healthy workplace environments that mitigate incivility. The significant differences in perceived workplace incivility between the two cultural contexts highlight the importance of culturally sensitive management practices. As noted by Earley and Mosakowski (2004), adapting leadership styles and communication approaches to fit cultural expectations can lead to more effective employee engagement and satisfaction. Additionally, fostering a culture of open communication, as suggested by Gelfand et al. (2013), can empower employees to voice concerns regarding incivility without fear of retribution.

Moreover, organizations should implement comprehensive training programs focused on cultural competence, as highlighted by Kirkman et al. (2006). These programs can equip employees with the skills necessary to navigate multicultural interactions, reducing misunderstandings that may lead to incivility. By integrating these practices, organizations can create an inclusive workplace culture that respects cultural differences and promotes psychological safety, ultimately enhancing employee morale and productivity (Singh, 2023). The study’s insights also encourage future research to explore how variations in incivility perceptions can inform the development of tailored interventions that address specific cultural needs, ensuring a respectful work environment across diverse populations.

### Limitations and Future Directions

Firstly, this study focused on participants with fixed daily duty routines, potentially limiting the generalizability of the findings to individuals in more flexible work environments. Future research should aim to include a broader spectrum of work settings to better understand the nuances of workplace incivility across varied contexts. Secondly, while employing a comprehensive daily diary design to capture participants’ experiences and responses, it’s acknowledged that some individuals might have exaggerated or underreported their encounters with workplace incivility. To address this, future studies could explore innovative measurement techniques, such as event sampling, where employees record incidents of incivility immediately as they occur, to provide more accurate and real-time data.

Third, this study specifically examined coworker incivility, potentially limiting the applicability of the findings to other forms of incivility, such as supervisor incivility. Future research should broaden its scope to encompass various sources of incivility within the workplace to gain a more comprehensive understanding of its impact across different hierarchical levels. Fourth, one limitation of this research is the need to include a larger number of subjects from additional cities in China, as well as a more diverse range of employee types. Finally, by asking participants whether they experienced each of the eight instances of rudeness, this study was unable to ascertain the frequency of these incidents. Consequently, there may be limitations such as restricted ranges, leading to diminished variance and potentially underestimation of effect sizes. Moving forward, it is recommended that researchers utilize measures incorporating frequency as a response format to provide more nuanced insights into the prevalence and impact of workplace incivility. Longitudinal research examining the long-term impacts of incivility on employee well-being in diverse teams would provide valuable insights. Understanding the sustained effects of workplace incivility on mental health and job satisfaction could lead to more effective strategies for addressing these issues. By exploring the intersection of cultural context, organizational policies, and employee well-being, future research can contribute to a more comprehensive understanding of workplace dynamics in a globalized world.

### Conclusions

The findings of this study suggest that the validated and culturally adapted scale of perceived workplace incivility can effectively measure perceived workplace incivility in both China and Pakistan. Through quantitative analysis of the test data, it was observed that the eight items displayed medium to high correlations with their total score. Furthermore, internal consistency reliability was confirmed with an Alpha Cronbach coefficient of 0.84 for the Chinese sample and 0.89 for the Pakistani sample, indicating strong reliability in both populations. Moreover, the correlation between item and structure validity was supported by the three-factor model, affirming the robustness of the scale. Consequently, it can be concluded that the workplace incivility scale demonstrates reliability for research purposes within service-providing organizations in both Chinese and Pakistani populations. Additionally, the measurement equivalence is confirmed in this study. In cross-cultural comparative studies, it is imperative to establish equivalent measurements for relevant constructs across different cultures. Without confirming this equivalence, making meaningful comparisons of results across countries becomes challenging.

## Data Availability

The datasets processed and analyzed during the current study are available from the corresponding/first author upon reasonable request.*
